# Partial characterization of a novel anti-inflammatory protein from salivary gland extract of *Hyalomma anatolicum anatolicum* 77Acari: Ixodidae) ticks

**DOI:** 10.14202/vetworld.2015.772-776

**Published:** 2015-06-24

**Authors:** Mayukh Ghosh, Nirmal Sangwan, Arun K. Sangwan

**Affiliations:** 1 Department of Veterinary Physiology and Biochemistry, College of Veterinary Sciences, Lala Lajpat Rai University of Veterinary and Animal Sciences, Hisar, Haryana, India; 2 Department of Veterinary Parasitology, College of Veterinary Sciences, Lala Lajpat Rai University of Veterinary and Animal Sciences, Hisar, Haryana, India

**Keywords:** anti-inflammatory, *Hyalomma anatolicum anatolicum*, size-exclusion chromatography, sodium dodecyl sulfate - polyacrylamide gel electrophoresis, tropical theileriosis

## Abstract

**Aim:**

*Hyalomma anatolicum anatolicum* ticks transmit *Theileria annulata*, causative agent of tropical theileriosis to cattle and buffaloes causing a major economic loss in terms of production and mortality in tropical countries. Ticks have evolved several immune evading strategies to circumvent hosts’ rejection and achieve engorgement. Successful feeding of ticks relies on a pharmacy of chemicals located in their complex salivary glands and secreted saliva. These chemicals in saliva could inhibit host inflammatory responses through modulating cytokine secretion and detoxifying reactive oxygen species. Therefore, the present study was aimed to characterize anti-inflammatory peptides from salivary gland extract (SGE) of *H. a. anatolicum* ticks with a view that this information could be utilized in raising vaccines, designing synthetic peptides or peptidomimetics which can further be developed as novel therapeutics.

**Materials and Methods:**

Salivary glands were dissected out from partially fed adult female *H. a. anatolicum* ticks and homogenized under the ice to prepare SGE. Gel filtration chromatography was performed using Sephadex G-50 column to fractionate the crude extract. Protein was estimated in each fraction and analyzed for identification of anti-inflammatory activity. Sodium dodecyl sulfate - polyacrylamide gel electrophoresis (SDS-PAGE) was run for further characterization of protein in desired fractions.

**Results:**

A novel 28 kDa protein was identified in *H. a. anatolicum* SGE with pronounced anti-inflammatory activity.

**Conclusion:**

Purification and partial characterization of *H. a. anatolicum* SGE by size-exclusion chromatography and SDS-PAGE depicted a 28 kDa protein with prominent anti-inflammatory activity.

## Introduction

Ticks are obligatory ectoparasites that exclusively feed on their host blood. *Hyalomma anatolicum anatolicum* tick is one of the most important ectoparasite of cattle and buffaloes with a wide host range because it acts as a vector of *Theileria annulata* causing tropical theileriosis [1,2]. Bovine tropical theileriosis, an unapparent infection of indigenous cattle and buffaloes, has emerged as one of the lethal disease of taurine cattle and their crosses especially due to large scale cross-breeding programs [3]. The estimated annual loss in terms of production and mortality in India by *T. annulata* alone accounts US$384.3 million [4].

Hence, control of tick infestation stipulates instantaneous consideration and intense research for improved livestock production. Ticks have evolved series of strategies to circumvent host defenses during their prolonged meal. Among them, secretion of saliva at the attachment site(s) possesses a critical role [5-7]. Despite the host’s armor of rejection mechanisms, the tick manages to remain attached and achieve engorgement due to the varied repertoire of pharmacologically active components in their salivary cocktail [8-17]. Related analysis of tick saliva have confirmed about the presence of active anti-inflammatory, antihemostatic/antithrombotic, and immune-modulatory proteins, extracted from saliva and salivary glands of different genera of ticks, that help these arthropods to combat host defense by lowering hosts’ B and T-cell responses, altering blood flow, inhibiting inflammation, and thus facilitate the pathogen transmission and infection [18-34].

However, to design effective tick control strategies and tick derived therapeutics, an elaborate knowledge about the tick sialome is required for further development of anti-tick vaccines or synthetic peptide based novel therapeutics. Thus, the present study deals with the proteomic approach to identify and characterize a novel anti-inflammatory protein from salivary gland extract (SGE) of *H. a. anatolicum* ticks.

## Materials and Methods

### Ethical approval

All the research experiments were conducted after approval of Institutional Animal Ethics Committee.

### Collection of ticks

Semi-fed adult female *H. a. anatolicum* ticks were randomly collected from buffaloes reared by farmers in unorganized cattle farms around the villages of Hisar district, Haryana, India.

### Tick dissection and collection of salivary glands

After washing with normal saline, collected ticks were glued to the bottom of a Petri dish with their dorsal surface upward. Ticks were incised along the dorsal-lateral margin by using fine scalpel blade under a stereoscopic dissection microscope (Magnus MSZ-TR). Non-infected salivary glands were removed by fine tip forceps and transferred into 0.1 M phosphate buffer saline (PBS) (pH 6.0) containing, 5% glycerol, protease inhibitor cocktail (Sigma, USA), and stored at −40°C until extract preparation [21]. The dissection was performed throughout under ice.

### SGE preparation

The tick salivary glands were homogenized under ice using tissue homogenizer (IKA T10 basic Ultra-Turrax) in 0.1 M PBS, pH 6.0, containing protease inhibitor cocktail. To remove the tissue debris and particulate materials, the homogenate was centrifuged at 10000 rpm for 15 min at 4°C. Then, the clear supernatant was collected and filtered through 0.22 µ syringe filter (MILLEX-GV) and stored at −40°C until further analysis.

### Protein purification by gel filtration chromatography

The SGE (400 µl) was applied to Sephadex G-50 (Sigma. USA) gel filtration column (1 cm × 16 cm) equilibrated with 0.1 M PBS, pH 6.0. Elution was performed with the same buffer, collecting fractions of 300 µl each. Chromatography was performed at cold chamber, and the column was calibrated with molecular-weight markers from Sigma (alcohol dehydrogenase, 150 kDa; albumin, 66 kDa; carbonic anhydrase, 29 kDa; cytochrome C, 12.4 kDa; and the void volume determined with Blue Dextran, 2000 kD). The approximate molecular weights of proteins were determined by interpolation with a standard curve of V_e_/V_0_ against log molecular weight.

### Estimation of protein concentration in fractions

The protein concentration in each fraction was estimated by the method of Lowry *et al*. [35] using bovine serum albumin (Hi-media) as standard.

### Anti-inflammatory activity assay

The red blood cell (RBC) membrane stabilization has been used as method to study the anti-inflammatory activity in crude extract and isolated fractions [36]. Blood was collected from healthy buffaloes in equal volume of sterilized alsever solution (2% dextrose, 0.8% sodium citrate, 0.5% citric acid, and 0.42% sodium chloride in water) and centrifuged at 3000 rpm for 15 min at 37°C. Packed cells were washed with isosaline (0.85%) and a 10% (v/v) suspension of RBC was made with the same. The assay mixture contained the SGEs (2, 4, 8, and 16 µg/ml protein concentrations), 1 ml of phosphate buffer (0.15 M, pH 7.4), 2 ml of hyposaline (0.36%) and 0.5 ml of RBC suspension. Instead of hyposaline, 2 ml of distilled water was used in the control. All the assay mixtures were incubated at 37°C for 30 min and centrifuged at 3000 rpm for 15 min at 37°C. The hemoglobin content in the supernatant was estimated from OD_560 nm_ using Ultraviolet-visible spectrophotometer (Thermo Scientific Multiskan^®^ Spectrum). The percentage of hemolysis was calculated using the following formula where hemolysis produced in control (distilled water) was assumed to be 100%.

% Hemolysis = OD of treated sample × 100/OD of control

The prevention of hemolysis was taken as a measure of anti-inflammatory activity.

### Characterization of peptides on Sodium dodecyl sulfate - polyacrylamide gel electrophoresis (SDS-PAGE)

The peptides in crude extract as well as fractions were further characterized by discontinuous SDS-PAGE (4% stacking and 15% resolving gel) using Laemmli buffer system [37] along with Broad Range Protein Molecular-weight Markers (Thermo Scientific, USA). To observe protein bands the gel was silver stained [38]. The molecular weight of peptide bands was interpolated with reference to the standard curve of marker proteins.

## Results

### Purification and partial characterization of peptides from tick SGEs

**Figure-1:**
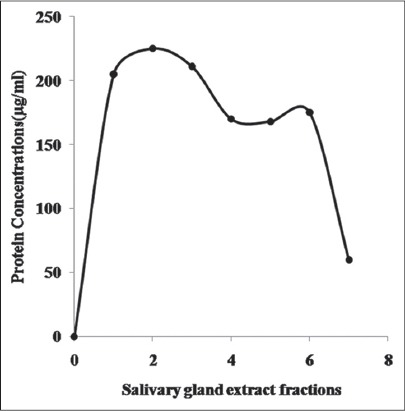
Fractionation of protein/peptides using Sephadex G-50 size-exclusion chromatography from *Hyalomma anatolicum anatolicum* tick salivary gland extract. Two peaks are centered on fractions 1 and 2 and fractions 5 and 6. Protein concentration in each of the fractions is also shown.

**Figure-2:**
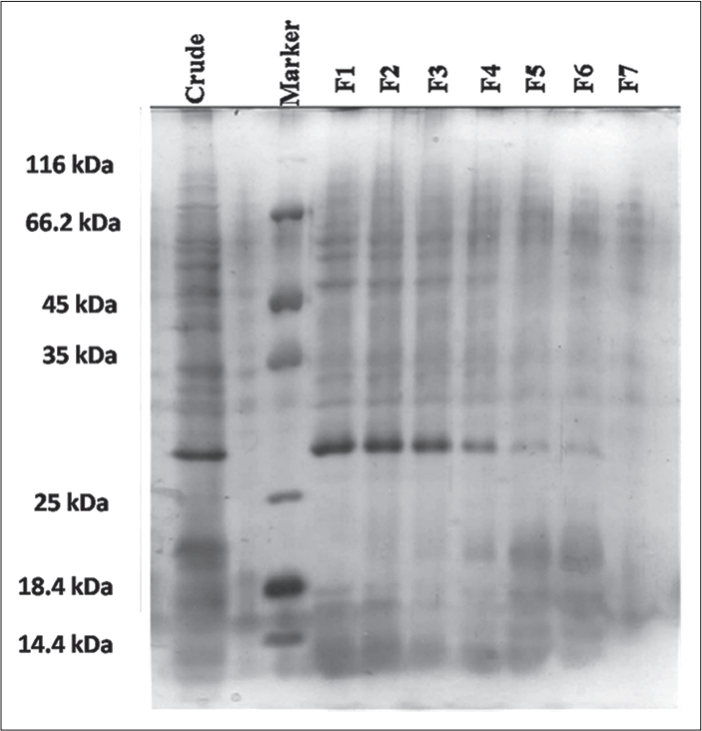
Protein profile of fractionated *Hyalomma anatolicum anatolicum* tick salivary gland extract characterized by Sodium dodecyl sulfate - polyacrylamide gel electrophoresis on silver stained 15% acrylamide gel slab. Lane 1 shows the protein profile of crude salivary extract of the tick; lane 3 is the protein molecular marker and the corresponding size of the marker proteins are stated sidewise; lane 4-10 show the protein profile of the gel filtration chromatography fraction 1-7 stated as F1-F7.

In preliminary characterization, the crude extract from the tick salivary glands have been fractionated by Sephadex G-50 gel filtration chromatography and protein concentration in each of these fractions is indicated in Figure-1. Electrophoretic pattern of the proteins/peptides in all the fractions shown in Figure-2 clearly depicts 20 prominent protein/peptide bands with molecular weight ranging between 10 kDa and 116 kDa. The number and type of peptide bands in first four fractions are similar with a distinct protein band of around 27.8 kDa which is getting very faint in the later fractions (fraction-5 onward). These fractions along with the crude extract were further analyzed for anti-inflammatory activity.

### Anti-inflammatory activity in crude extract and isolated fractions

**Figure-3:**
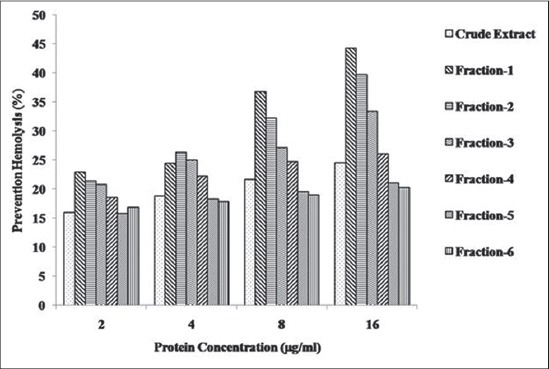
Anti-inflammatory activity in crude salivary gland extracts and gel filtration chromatography fractions of *Hyalomma anatolicum anatolicum* ticks at different protein concentration.

The anti-inflammatory activity was increased with the increase in protein concentration in crude SGEs as well as in fractions. But it did not follow the linear correlation. In *H. a. anatolicum* crude SGE, the anti-inflammatory activity increased from an initial mean value of 15.99±0.54% to final value of 24.6±0.64% with the increase in protein concentration from 2 µg/ml to 16 µg/ml. The anti-inflammatory activity of the fractions showed similar results (Figure-3). In fraction-1, the anti-inflammatory activity increased from an initial value of 22.98% to final value of 42.25% with the increase in protein concentration from 2 µg/ml to 16 µg/ml whereas in fraction-6 it increased from an initial value of 16.92% to final value of 20.26%. Fraction-1 showed significantly (p<0.05) higher activity than fraction-6 at all the different protein concentrations taken which correlates well with the protein (27.8 kDa) concentration shown in SDS-PAGE analysis (Figure-2).

## Discussion

The anti-inflammatory activity reported in fraction-1 to fraction-4 of *H. a. anatolicum* SGE is in accordance with the sialomic analysis in related tick genera, where the conservation of the activity was depicted to satisfy biological demand of the ticks [13,21,24,39-42]. The fractions having anti-inflammatory activity showed a positive correlation with the concentrations of total protein in those fractions. All the fractions having anti-inflammatory activity had the common protein with molecular weight of 27.8 kDa. Furthermore, the electrophoretic profile revealed more pronounced protein band in fraction-1 having anti-inflammatory activity as compared to the bands in fraction-5 and 6 where the activity was less. The protein was almost absent in fraction-7 with negligible activity. The SDS-PAGE also showed the low molecular weight peptide bands which appeared in the first four fractions and could be due to denaturation of multimeric proteins which have got denatured during SDS treatment. It has been reported that the anti-inflammatory activity in the tick *Ixodes scapularis*, the vector of Lyme disease or Borreliosis, to affect the host proteolytic activity in the sites of infestation was mainly through the release of sialostatin L inhibited proliferation of cytotoxic T lymphocytes [20]. It has been found that Borrelia pathogens exploit tick saliva to facilitate their transmission by repressing host innate immunity and cutaneous inflammation [40]. The presence of two families of immunomodulatory peptides (hyalomin-A and -B) were identified by Wu *et al*. [21] from the salivary glands of the hard tick *H. a. asiaticum* having molecular weights of only 1231.16 and 3688.2 Dalton. These two peptides were also found to suppress host inflammatory response by modulating cytokine secretion and detoxifying reactive oxygen species. Hence, the anti-inflammatory activity may have a relation with other modes of defense mechanisms in ticks. The experiment of Vancova *et al*. [39] and Déruaz *et al*. [14] depicted the presence of Evasin-3 and Evasin-4 like anti-chemokine activity in SGEs of *Rhipicephalus sanguineus* ticks. Evasin-3 was found to be important in controlling neutrophils during blood-feeding and its conservation among metastriate ixodid tick species. Use of molecular dynamics simulations depicted a competition for histamine binding between hosts’ native histamine receptor and the secreted tick salivary lipocalin where histamine choose the later with more affinity [28]. Several members of serine protease inhibitor (serpin) were identified in *Rhipicephalus* (*Boophilus*) Microplus, which are essential for blood coagulation, fibrinolysis, inflammation, and complement activation [31]. Proteomic and transcriptomic insight into *Dermacentor andersoni* saliva also explored numerous bioactive molecules including inflammation inhibitors [43]. Anti-inflammatory activity was also observed in *Ixodes ricinus* saliva where serpin IRS2 inhibits Th17 differentiation and impairs interleukin-6 (IL-6)/STAT-3 signaling pathway [44]. Serpins were also identified and analyzed in *Amblyomma americanum*, both male and female ticks. The female serpins were found to be conserved across taxa [45]. This related knowledge about tick sialome provides an overview about the conservation and potential of tick salivary anti-inflammatory proteins across the genera, modulating host immune response. It also corroborate with our finding about *H. a. anatolicum* salivary anti-inflammatory activity in the recent study. These also provide an account of versatility and redundancy of pharmacologically active candidates in tick salivary cocktail and indicate that the anti-inflammatory activity may also have role in saliva activated pathogen transmission of *T. annulata* by *H. a. anatolicum* ticks as future prospect of the present study. Though it was a preliminary study regarding *H. a. anatolicum* sialome, further research is needed in this field to enlighten the mechanisms and pathways of these pharmacologically active salivary gland proteins which will be helpful in controlling ticks and tick-borne diseases.

## Authors’ Contributions

NS designed the experiment, data analyses and final corrections of the manuscript. MG and AKS collected the sample. AKS identified the tick species and contributed in the dissection of ticks. MG performed the dissection of ticks, wet lab analyses, and data analyses. All authors participated in manuscript preparation. All authors read and approved the final manuscript.
